# A Practical Model Evaluating Antiviral Cytokines by Natural Killer Cells in Treatment Naïve Patients with Chronic Hepatitis B Virus Infection

**DOI:** 10.1038/s41598-017-06192-1

**Published:** 2017-07-19

**Authors:** Xiaoyan Li, Yurong Gu, Xiaobo Guo, Lin Gu, Liang Zhou, Xiaojuan Wu, Xueqin Wang, Zania Stamataki, Yuehua Huang

**Affiliations:** 1 Department of Infectious Diseases, The Third Affiliated Hospital of Sun Yat-sen University, Guangzhou, China; 2 Department of Statistical Science, School of Mathematics, Sun Yat-Sen University, Guangzhou, China; 3 Southern China Center for Statistical Science, Sun Yat-Sen University, Guangzhou, China; 4 Department of Ophthalmology, University of Melbourne, Melbourne, Australia; 5 Guangdong Provincial Key Laboratory of Liver Disease Research, The Third Affiliated Hospital of Sun Yat-sen University, Guangzhou, China; 6 Zhongshan School of Medicine, Sun Yat-Sen University, Guangzhou, China; 7 Institute for Immunology and Immunotherapy and NIHR Biomedical Research Centre, University of Birmingham, Birmingham, United Kingdom

## Abstract

Natural killer (NK) cells play a major role in anti-viral immunity as first line defense during hepatitis B infection, particularly in untreated patients whose T cells functions are profoundly impaired. Cytokine interferon (IFN)-γ and tumor necrosis factor (TNF)-α produced by NK cells are important anti-viral factors. However, there is lack of a quantifiable model to evaluate cytokine responses by NK cells. In this study, almost half of the patients (47.9%) beyond treatment criteria had high cytokine activity, although it was lower than those recommended for antiviral therapy (78.2%). Moreover, we developed a model that low levels of HBsAg, HBcAb, and albumin and high fibrosis values predicted strong antiviral cytokine production by NK cells. Based on the cut-off score (0.361) obtained from the multivariable model, patients with 67%, 8%, 92%, and 74% in immune-active (IA), immune-tolerant (IT), immune-inactive (IC), and grey zone (GZ), respectively, showed active antiviral cytokines produced by NK cells. These results suggest that those who possess activated cytokine responses beyond the current treatment criteria may have potential implications for the timing of antiviral therapy to achieve better virus control.

## Introduction

Despite the availability of a prophylactic vaccine, chronic hepatitis B (CHB) affects nearly 350 million people worldwide^1^. For those affected, lifelong infection carries the risk of developing cirrhosis, hepatocellular carcinoma (HCC), and liver failure. Efforts to understand the immunopathogenesis of CHB focus on the intricate relationship between the virus and the host. The natural course of this relationship progresses predictably through the following four recognized sequential phases: immune tolerant (IT); immune active (IA); inactive carrier (IC); and hepatitis B e antigen (HBeAg)-negative immune reactivation^2^. Although this phase-focused view of CHB allows for the design of rational therapeutic practices, the distinctions among the phases of infection are not absolute^3^.

Current treatment guidelines recommend the presence of active liver inflammation on histology or elevated serum levels of alanine aminotransferase (ALT) of more than 2 upper limit of normal (ULN) level plus elevated HBV DNA above 2000 IU/mL (HBeAg negative) or 20,000 IU/mL (HBeAg positive) as criteria for initiating antiviral therapy^4^. However, an area of ongoing debate in CHB is the management of certain subgroups of patients beyond this treatment threshold, including patients with ALT < 2 x ULN, those in the immune-tolerant stage, patients with intermittently elevated ALT that can escape typical clinical surveillance, and those in the so-called “inactive carrier stage”^5^. There are no formal treatment recommendations for these patients, although a liver biopsy would be considered for some of them. These patients are believed to either lack an HBV immune response or have a favourable prognosis and, therefore, are not considered treatment candidates^6–8^. Indeed, HBV-specific T-cell reactivity is weak either due to T-cell exhaustion or viral escape^9–14^. Nevertheless, this does not fully explain why the immune system is sometimes able to either control infection or induce liver injury^15^. Moreover, some studies challenge the dogma of defective HBV-specific T cells in younger immune-tolerant patients. Up to 37% of CHB patients with normal ALT already have significant necrotic inflammation, fibrosis and even cirrhosis on liver biopsy^2, 16–18^.

Natural killer (NK) cells are a major component of the innate immune system and perform crucial functions in defence against viral infection and cancer^19–21^. NK cells produce interferon (IFN)-γ and potent cytotoxic effector cells to mediate immunity against HBV infection^22^. Based on their expression of CD56, NK cells are classified into NK^dim^ and NK^bright^ subsets. NK^bright^ cells are primary cytokine producers, while NK^dim^ cells mainly perform cytotoxic functions in the periphery^23–27^. NK cells are also reported to boost the efficacy of PEGylated interferon-α in CHB treatment, which correlated with peak virological responses^28^.

A systematic review and multiple studies have identifies the phenotype and function of NK cells in CHB patients. Several studies have found that impaired IFN-γ from NK cells in CHB contributes to viral persistence^29, 30^. Other studies suggested that NK cells can exhibit activated phenotypes that are associated with viral clearance^31, 32^. Recent findings indicated their immunomodulatory function, suggesting regulatory roles for NK cells in other immune cells^26, 33^. However, these studies have limitations: (1) There are no detailed comprehensive comparisons of cytokine functions in NK cells between CHB patients who are strongly recommended for initiation of routine antiviral therapy and those who are beyond the treatment criteria according to current HBV guidelines from international associations; (2) there are inadequate data to evaluate the correlation between NK-cell driven antiviral cytokines and clinical-virological parameters; and (3) there is lack of a quantifiable model to calculate cytokine responses by NK cells in CHB patients.

In our current study, we, therefore, performed a comprehensive investigation of NK cell frequency, phenotype, and cytokine production in a large cohort of well-characterized CHB patients. We displayed an overall picture of NK cell production of antiviral cytokines in CHB patients who are beyond the treatment regimen criteria (CAN) compared to those who satisfy the recommendations (CA) for antiviral therapy. The relevance of cytokine expression and a broader repertoire of clinical-virological variables were clarified. A mathematical model with unique specificity and sensitivity was generated to evaluate the innate immunity. This report provided a feasible and quantifiable predictor to identify the efficiency of NK cell antiviral activity in CHB patients.

## Materials and Methods

### Subjects

Consecutive adult patients with CHB infection observed in the dedicated viral hepatitis clinic of the Third Affiliated Hospital of Sun Yat-sen University between July 2015 and July 2016 were recruited. Patients who received antiviral treatment (IFN-α or nucleoside analogues) within the previous 6 months; patients with human immunodeficiency virus (HIV), hepatitis C virus, or hepatitis D virus coinfection; and patients with end-stage liver insufficiency, autoimmune disorders, immunosuppressive treatment, cirrhosis, and malignancies were excluded. The study was approved by the Institutional Review Board of the Sun Yat-sen University. Informed consent has been obtained from each patient involving in this study. All methods were performed in accordance with the relevant guidelines and regulations.

Of the 226 individuals eligible for participation in this study, 33 were excluded because of missing values, leaving 193 patients available for analysis. The classification and denomination of the patients with CHB in this work were based exclusively on serologic and biochemical parameters in accordance with published international treatment guidelines as follows: (1) inactive carriers (IC): normal ALT level, HBeAg antibody (anti-HBeAg) positive, and low HBV DNA level; (2) chronic active hepatitis with recommendation for initiation of antiviral therapy (CA): elevated ALT level, HBeAg positive or negative; and (3) CHB infection without a strong recommendation to receive antiviral therapy (CAN), including patients during the immune-tolerant phase and those who are in the “grey zones”, meaning that ALT and HBV DNA levels are not in the same traditionally characterized phases^4^. Blood was also obtained from age-matched non-HBV infected healthy controls (n = 16). Information on the demographics (age range, sex distribution), body mass index (BMI), HBV markers (HBeAg, HBV DNA, HBV surface antigen [HBsAg], anti-HBeAg, antibody to HBV core antigen [HBcAb]), hepatic panel (albumin [ALB], ALT, aspartate aminotransferase [AST], total bilirubin [TBIL]), HBV genotypes, and liver stiffness measurement (Fibroscan value) is listed in Table 1.

**Table 1:** Clinical-virology Characteristics of Patients Included in the Study.

Characteristics	CA (n = 46)	CAN (n = 128)	*p* value^a^
Age, y			
Median (25%CI, 75%CI)	28.5(25,33.75)	29(25,35)	0.9604
Sex, n (%)			1.0000
Female	14(29.2)	44(30.6)	
Male	34(70.8)	100(69.4)	
Body mass index			
Median (25%CI, 75%CI)	20.55(19.08,22.71)	21.01(19.14,23.04)	0.7647
HBV DNA, log 10IU/ml			
Median (25%CI, 75%CI)	7.943(6.765,8.23)	4.483(3.421,8.23)	0.0000
HBV genotype, n (%)			0.0019
B	28(58.3)	81(56.2)	
C	18(37.5)	28(19.4)	
Other	2(4.2)	35(24.3)	
HBeAg status, n (%)			0.0003
HBeAg negative	12(25)	81(56.2)	
HBeAg positive	36(75)	62(43.1)	
Missing	0(0)	1(0.7)	P020
HBsAg, IU/ml			
Median (25%CI, 75%CI)	12260(2766, 37740)	2894(1219, 28550)	0.0072
HBcAb level, COI			
Median (25%CI, 75%CI)	0.008(0.005, 0.009)	0.009(0.008, 0.01)	0.0000
Smoker, n (%)			0.8890
No	44(91.7)	129(89.6)	
Yes	4(8.3)	15(10.4)	
Vertical transmission, n (%)			1.0000
No	36(75)	108(75)	
Yes	7(14.6)	21(14.6)	
Missing	5(10.4)	15(10.4)	
ALT, U/L			
Median (25%CI, 75%CI)	108(76, 196.5)	28(22, 37)	0.0000
TBIL, mg/dl			
Median (25%CI, 75%CI)	15.8(13.1, 21.7)	12.15(9.425, 15.75)	0.0001
ALB, g/l			
Median (25%CI, 75%CI)	44.7(41.9, 46.1)	46.4(44.8, 48.1)	0.0062
FibroScan value, Kpa			
Median (25%CI, 75%CI)	6.35(5.375, 10.95)	4.8(4.2, 5.6)	0.0000

CI: Confidence interval, other: HBV genotype B and C mixed or indeterminate, COI: Cutoff index.

The p-values were calculated using Mann-Whitney test for continuous variables and the χ^2^ test for categorical variables.

### Phenotypical and Intercellular Staining and Flow Cytometry Analysis

Peripheral blood mononuclear cells (PBMCs) were isolated from fresh blood samples using Ficoll density gradients as described previously^34^. Isolated PBMCs were stained for surface markers, fixed, permeabilized with IntraPreReagent (Beckman Coulter, Fullerton, CA), and further stained with antibodies directed against intracellular markers. Leukocytes were stimulated with Leukocyte Activation Cocktail (BD Bioscience, San Jose, CA, USA) at 37 °C for 4 h prior to the intracellular staining using Pharmingen’s staining protocol. Anti-human mAbs against CD3-PE-CF594, CD56-FITC, IFN-γ-PE, and TNF-α-PE with corresponding isotype-matched controls were purchased from BD Biosciences (San Jose, CA, USA). Data were acquired on a Gallios instrument (Beckman Coulter, Brea, CA, USA) and analysed with FlowJo software (FlowJo, LCC, USA).

### Clinical and Serologic Parameters

Upon recruitment to the study, serum was tested for HBsAg, anti-HBsAg, anti-HBeAg, HBeAg, and HBcAb using commercial kits (Abbott Laboratory, North Chicago, IL). HBV genotype was performed by direct sequencing. Quantitative HBsAg was measured by the Elecsys HBsAg II Quant reagent kits (Roche Diagnostics, Indianapolis, IN) according to the manufacturer’s instructions. HBcAb levels were quantified with a chemiluminescence immunoassay (Roche Diagnostics, Indianapolis, IN). Serum HBV DNA level was measured by Roche COBAS Ampliprep/COBAS TaqMan HBV Test v2.0 (dynamic range from 20 to 1.7E + 08 IU/mL), (Roche Molecular Diagnostics, Branchburg, NJ). Fibrosis levels were defined by liver stiffness measurements (Fibroscan, Echosens, Paris, France).

### Statistical Analysis

We compared two patient groups using the Mann-Whitney test for continuous variables and the χ^2^ test for categorical variables. We explored the association between two continuous variables using a linear regression model and Pearson correlation. The association between the continuous and categorical variable was used the Kruskall-Wallis test. For the cluster analysis, we used the K-Means method to separate the sample into two clusters based on the levels of anti-viral cytokines on total NK cells, NK^dim^ cells and NK^bright^ cells. A multivariate logistic regression model was employed to select the combination of clinical variables associated with the two identified clusters. Stepwise variable selection with AIC criteria was used to select the important clinical variables. The logistic regression coefficients were used to generate nomograms. Discrimination was evaluated by analysing the area under the receiver operating characteristic curve (ROC curve). We used the Youden Index to generate the optimum cutoff for the clinical variables. The nomogram was created using the rms package of R software. All the other statistical tests were performed using R software version 3.2.2. Statistical significance was set to 0.05. We used Bonferroni correction to correct for the multiple comparisons.

## Results

### Peripheral Blood NK Cell Frequency and Subsets Distribution in CHB Patients

**Figure 1:**
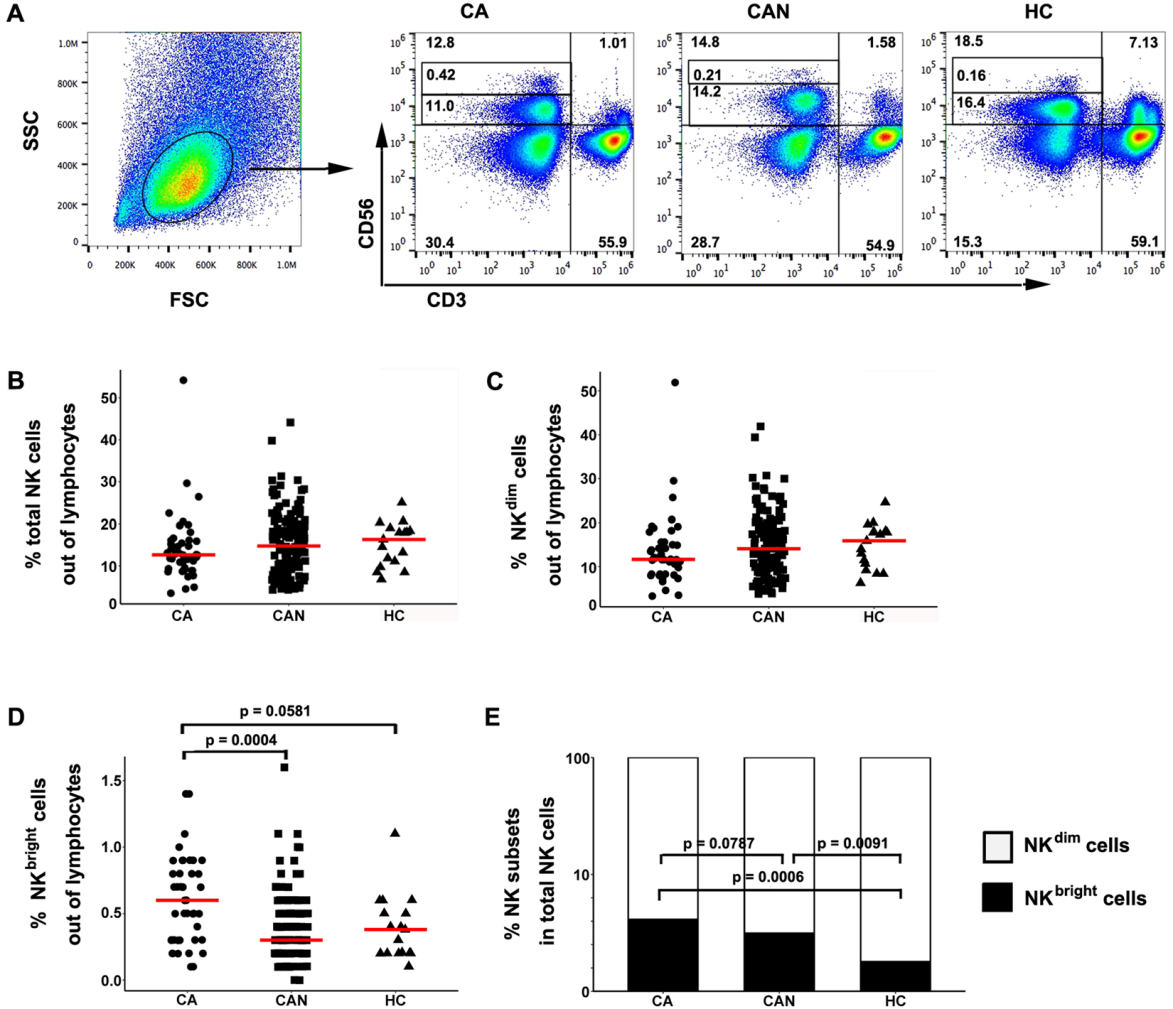
NK cell gating strategy and population frequency analysis in CHB patients and healthy donors. (**A**) NK cells and subsets were derived from total live PBMCs gated by forward and side scatter followed by the CD56^+^CD3^−^ phenotype. NK^dim^ and NK^bright^ cells are defined by the expression of CD56 intensity. The values in the left upper quadrants represent the percentages of total NK, NK^dim^ and NK^bright^ cells among total lymphocytes. The data shown are representative of at least ten individuals from more than three independent experiments. (**B–D**) The frequency of total NK, NK^dim^ and NK^bright^ cells in CHB patient groups. Values are displayed as a percentage of NK cells and subsets among total lymphocytes. Each symbol denotes an individual subject. (E) Summary bar charts of the distribution of NK^dim^ and NK^bright^ subsets depicted as the frequency of total NK cells. CA, CAN: CHB patients who are within or beyond current treatment guidelines, respectively.

We characterized NK cell frequency and subsets in 46 and 128 CHB patients within (CA) or beyond (CAN) current treatment guidelines, respectively. The frequency of the circulating NK cells and the NK^dim^ subset tended to be lower in CHB patients than in healthy subjects, while the lowest proportion was observed in the CA group, even though both patient groups failed to show statistically significant differences from healthy subjects. Instead, the NK^bright^ subpopulation was increased in CA compared with CAN patients (*p* = 0.0004) and healthy subjects (*p* = 0.0581 (Fig. 1D). This increase in the frequency of NK^bright^ cells seemed to be reflected in the subset distribution, suggesting a shift in the NK cell compartment towards more CD56^bright^ cells, which are primary cytokine producers, from healthy controls to CAN, achieving the greatest proportions in CA patients (Fig. 1E).

### NK-cell Expressing Cytokines in CHB Patients in Different Stages

**Figure 2:**
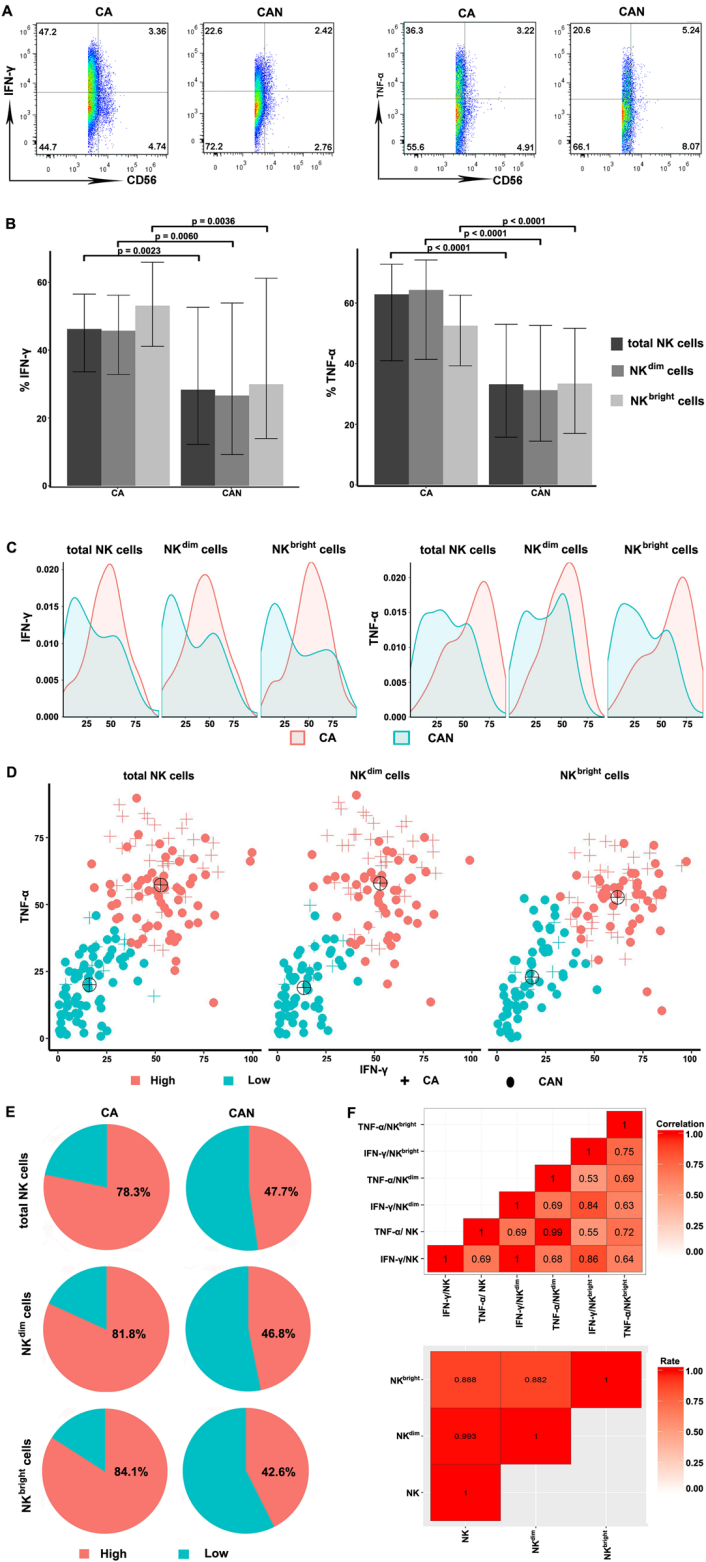
Quantification of cytokine productions by NK cells and their subsets in CHB patients. (**A**) FACS plot showed the representatives of IFN-γ and TNF-αproduced by NK cells in CA and CAN groups. (**B**) The production of IFN-γ and TNF-αby NK cells and their subsets were compared between patients in CA and CAN groups and healthy controls. The comparisons were using Mann-Whitney test. (**C**) Overlapping sections of cytokine distribution signify the proportion of patients with similar abilities to produce cytokines in the two patient groups. (**D**) Clustering of CHB patients with IFN-γ and TNF-α produced by total NK (left), NK^dim^ (middle) and NK^bright^ (right) cells using the K-means method. Black targets represent the cluster centres. The high or low levels of IFN-γ and TNF-α clusters are shown in red or blue colours, respectively. The crosses and circles represent CA and CAN patients, respectively. (**E**) Pie charts show the percentage of total NK, NK^dim^ and NK^bright^ cells able to produce high (red) or low (blue) levels of cytokines in the indicated categories of CHB patients. (**F**) The Pearson correlation coefficient between IFN-γ and TNF-α from total NK, NK^dim^ and NK^bright^, respectively (upper). The concordance rate of clustering results based on IFN-γ and TNF-α levels from total NK, NK^dim^ and NK^bright^, respectively (lower). Pseudocolours indicate correlation levels from low (0) to high (1), ranging from a weak (white) to strong (red) association strength.

To investigate whether CHB patients beyond current treatment criteria are characterized by a state of defective antiviral capacity, we analysed two major effector cytokines, IFN-γ and TNF-α, produced by NK cells in CA and CAN patients. As expected, the levels of both IFN-γ and TNF-α produced by total NK cells were higher in patients who were classified as chronically active (mean values, 46.2 for IFN-γ; and 62.9 for TNF-α) compared with the CAN cohort (28.3 for IFN-γ (*p* = 0.0023) or 33.2 for TNF-α (*p* < 0.0001)). Similar results were observed in the proportions of NK cell subsets producing IFN-γ and TNF-α between the two distinct patient groups (Fig. 2B). Moreover, IFN-γ production was lower in the NK^dim^ subset regardless of the CA or CAN status compared to the NK^bright^ subset of CAN patients, reaching the highest level in NK^bright^ cells of the CA group. We also defined the cytokine distribution in NK cells and their subsets. The data indicated that the overlapping sections of IFN-γ and TNF-α in both patient groups were 70.4%, 71.4%, and 65.4% for IFN-γ and 62.9%, 59.6% and 69.8% for TNF-α from NK, NK^dim^, and NK^bright^ cells, respectively, although their peak distinctions were clear (Fig. 2C).

Because HBV infection has a significant impact on IFN-γ and TNF-α, we further investigated whether the combined evaluation of IFN-γ and TNF-α could better categorize the maturation of an efficient antiviral immune control in CHB patients. K-means analysis was performed to differentiate high (High group) and low (Low group) antiviral immunity within the groups of CA and CAN based on the combined expression of the two cytokines (Fig. 2D). Almost half of the patients (47.9%) in the CAN group had high NK cytokine production activity, although it was lower than that (78.2%) in the CA group. Similar observations between the CA and CAN groups were found when using NK^dim^ and NK^bright^ cells to classify homogeneous clusters (Fig. 2E). Finally, the correlation between IFN-γ and TNF-α in the current CHB cohort indicated that these 2 parameters were highly consistent. Likewise, there was a direct correlation between NK cells and their subsets (Fig. 2F).

Taken together, these results show that a certain number of CHB patients who are beyond the current treatment guidelines still produce antiviral cytokines.

### Biochemical, Fibrosis, and Virological Correlates of NK Cell Subsets and Cytokine Profiles

**Figure 3:**
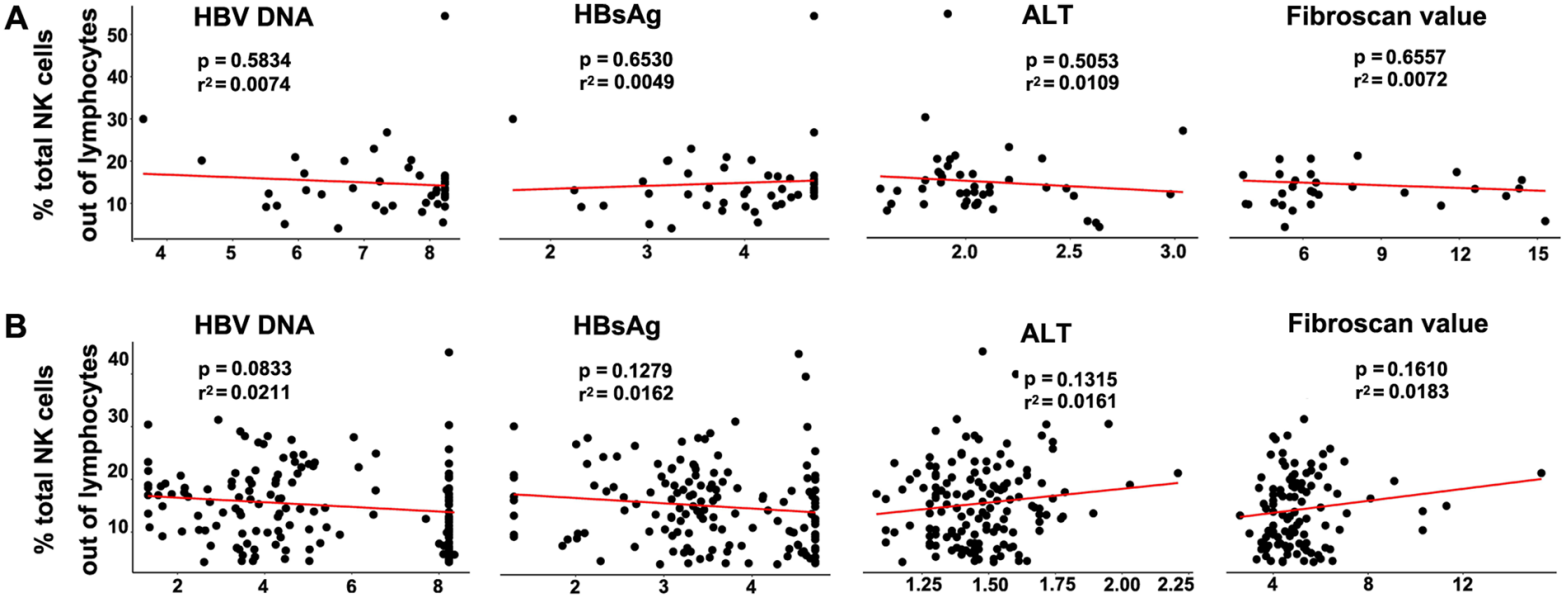
Correlations of clinical virology parameters with percentages of NK cells in CHB patients. Correlations of levels of HBV DNA, HBsAg, ALT and fibrosis, respectively, with NK frequency in patients in CA (**A**) and CAN (**B**) groups. Linear regression was used to explore the correlations. The fitted curve, *p* value and r^2^ were shown.

Several possible statistical correlations were sought between the frequency and cytokine profiles of NK cells and biologic (ALT), fibrosis (fibroscan value), or virological (HBV DNA, HBsAg) indicators. We found higher numbers of NK^bright^ cells correlated with higher HBV DNA levels, which were limited to the CA group (r^2^ = 0.0903; *p* = 0.0502) (Supplementary Figure 1). Additionally, there was a positive correlation between the frequency of NK^bright^ cells and fibrosis severity, which was confined in the CAN patients (r^2^ = 0.0687; *p* = 0.0064) (Supplementary Figure 2). Otherwise, no correlation was found between the HBsAg levels and ALT and the frequency of NK or NK^dim^ cells in both the CA and CAN groups (Fig. 3, Supplementary Figures 1, 2).

**Figure 4:**
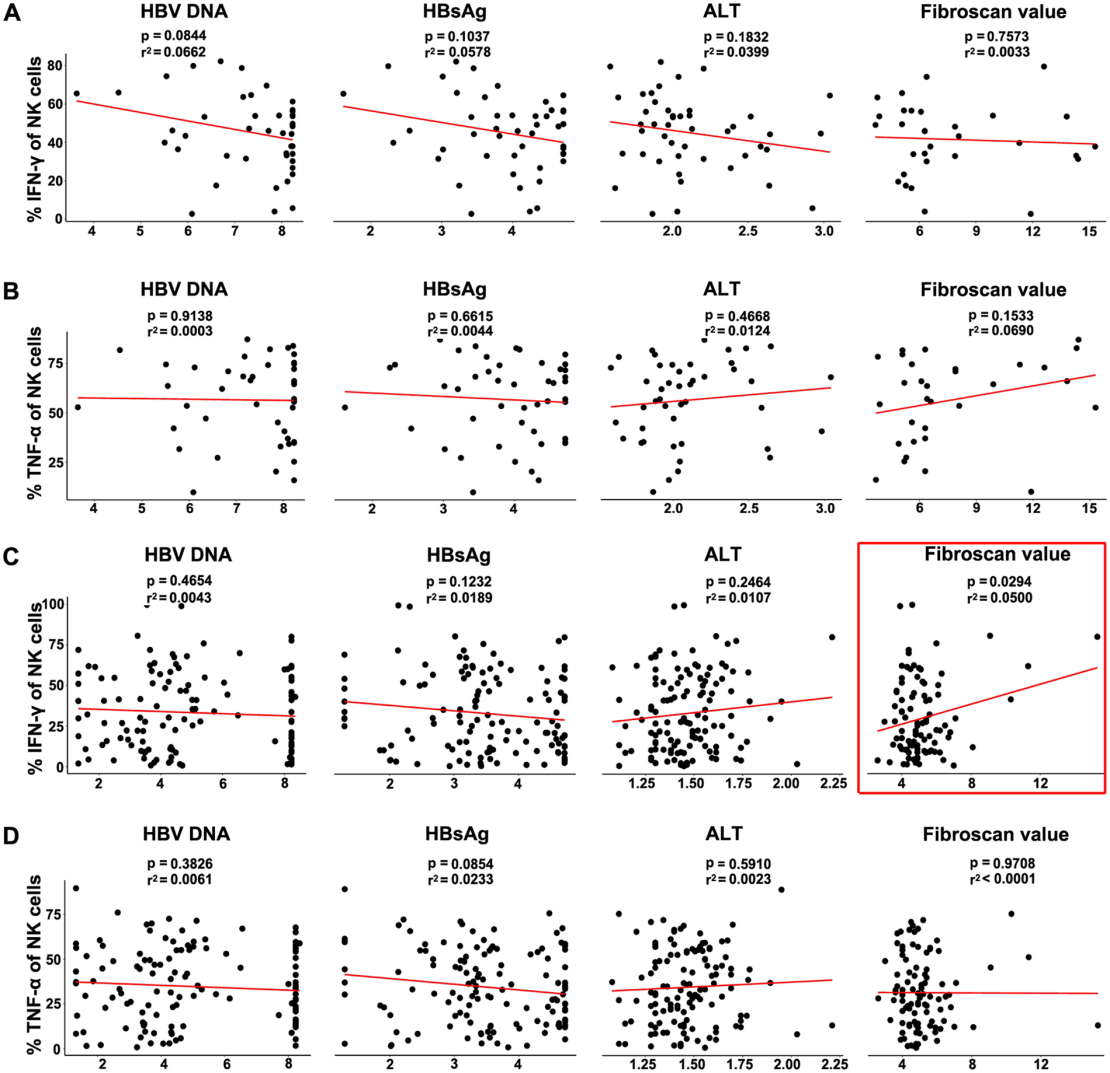
Correlations of clinical virology parameters with NK-cell-driven cytokine expression in CHB patients. Correlations of levels of HBV DNA, HBsAg, ALT and fibrosis, respectively, with levels of IFN-γ and TNF-α in patients in the CA (**A,B**) and CAN (**C,D**) groups. Linear regression was used to explore the correlations. The fitted curve, *p* value and r^2^ were shown.

We then dissected the association between the clinical-virological factors and the cytokines from NK cells and their subsets. Direct correlations between NK cells and their subsets exhibiting IFN-γ with fibrosis value were detected in the CAN group but not in the CA group (r^2^ = 0.0500, *p* = 0.0294; r^2^ = 0.0913, *p* = 0.0072; and r^2^ = 0.1193; *p* = 0.0021; for groups of total NK, NK^dim^, and NK^bright^ cells, respectively) (Fig. 4C, Supplementary Figures 5, 6). However, no correlation was found between the levels of ALT, HBV DNA, and HBsAg and cytokine production by NK cell populations of either CA or CAN patients (Fig. 4, Supplementary Figures 3–6).

### Simplified Model to Predict NK-cell Expressing Cytokine Profile

**Figure 5:**
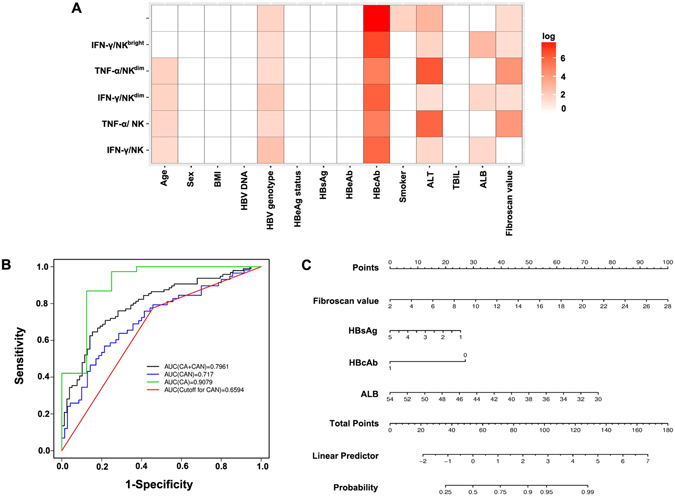
Development of prediction model of NK-cell cytokine function. Correlation of NK cells and their subsets expressing IFN-γ and TNF-α with 14 clinical virology characteristics. *p* < 0.05 was shown in colour and the depth of red colour represents the degree of significant difference. Pearson correlation was used for continuous clinical variables, and Kruskall Wallis test was used for categorical clinical variables. (**B**) ROC analysis. The ROC curves by these four clinical variable-based classifiers in all CHB patients (CA + CAN), CAN, CA patients, and the ROC curve by the cut-off score based classifier in CAN patients. (**C**) A visual nomogram used to assign the ability of NK-cell cytokine production by summing the scores of the above selected clinical-virological parameters shown on the point scale. The total score projected to the corresponding scale indicates the likelihood of active cytokine immunity. ROC, receiver operator characteristic; AUC, area under the curve.

To further evaluate the impact of the clinical-virological factors on the frequency and cytokine-producing function of the NK cell subsets, a multivariate logistic regression model, extended to 14 variables readily available in clinical practice, was used to predict the functional response of NK-cells in CHB. Stepwise variable selection with AIC criteria was used to select the important clinical variables. Four clinical parameters (Fibrosis value, HBsAg, HBcAb, ALB) were selected in the final model (Table 2). We then calculated the probability for the risk of High group for each patients based on the four selected clinical variables. The prognostic accuracy of the 4 clinical variables based classifier was assessed by using the ROC analysis, where the AUCs were 0.7961 in the CAN plus CA patients, 0.7170 in the CAN cohort and 0.9079 in the CA cohort, indicating appealing prognostic accuracy for the classifier. By using the Youden Index to generate the optimum cut-off score, we include those patients with a probability of 0.361 or higher in the group of patients with activated NK cell cytokine exhibiting function, and those with a probability lower than 0.361 in the group of patients with blunted expression of NK-cell driven cytokines (Fig. 5C). The AUC for the classifier based on the optimum cut-off was 0.6594 based on the sensitivity and specificity (Fig. 5B). To provide the clinician with a quantitative method to predict a patient’s probability of innate immunity prediction in CHB, we constructed the nomogram (Fig. 5C).

**Table 2:** Parameters Included in the Logistic Regression Model.

Parameters	Estimate	Std. Error	Z value	Pr(>|z|)
Intercept	11.43725	4.760678	2.402442	0.016286
FibroScan value	0.238008	0.125953	1.88965	0.058805
HBsAg	−0.446	0.204304	−2.183	0.029036
HBcAb	−1.66391	0.410519	−4.05318	5.05E-05
ALB	−0.23183	0.093025	−2.49215	0.012697

### A Threshold of NK-cell Function for Predicting Innate Virological Responses

Given the dynamic nature of CHB, the current CHB cohort plus an additional 19 patients who were in the inactive CHB phase (IC) were assessed by the above model to predict their innate cytokine functions. Therefore, the 193 patients were categorized into 4 phases as follows: IT phase (n = 13), IA phase (n = 103), IC phase (n = 19), and grey zone (GZ, meaning that HBV DNA and ALT levels do not fall into the same phase, n = 58). Patients in each phase were segregated into 2 groups with either active or inactive NK cell cytokine function based on the individual estimates derived from the nomogram. Table 3 showed the characteristics of each group with the predictors that were entered the model. The data showed that 67% and 34% patients in the IA phase were segregated into either high or low NK-cell cytokine production categories, respectively. Likewise, patients were segregated into high or low NK-cell cytokine production categories at rates of 8% and 92% in the IT phase, 26% and 74% in the IC phase and 45% and 55% in the GZ phase, respectively. Interestingly, we re-examined NK cell cytokine activity in the CA and CAN groups using this predictive model and found that 82.6% and 45.3% patients in the CA and CAN groups, respectively, were assigned to the high innate immunity category. These proportions of high cytokine function determined by the current prediction model were strongly in accordance with the exact cytokine levels determined by flow cytometry.

**Table 3:** Evaluation of NK Immunity Based on Baseline Clinical Parameters According to NK Score in CHB Patients with Different Stage.

Groups	Total No.		≥0.361 (n, %)	<0.361 (n, %)	AUC	Sensitivity	FPR
IA	103		69, 66.99	34, 33.01	0.810315431	0.855072464	0.382352941
IT	13		1, 7.69	12, 92.31	0.916666667	1	0.166666667
IC	19		5, 26.32	14, 73.68	0.514285714	0.6	0.642857143
GZ	58		26, 44.83	32, 55.17	0.717548077	0.846153846	0.625
CA	46		38, 82.61	8, 17.39	0.907894737	0.973684211	0.375
CAN	128		58, 45.31	70, 54.69	0.716995074	0.775862069	0.457142857

Overall, the current predictive nomogram revealed distinct NK cell cytokine function in CHB patients in different disease stages and clearly separated individuals with high innate cytokine activity from those with low activity although they might be in the same phase of CHB infection.

## Discussion

In the present study, we performed detailed and comprehensive analyses to determine the innate immunity of patients with CHB who meet the stringent inclusion (CA) or exclusion (CAN) criteria of the treatment guidelines. We showed the following: (1) Nearly half of the CAN patients preserved similar levels of cytokine expression by NK cells as those in the CA group, although the average level was lower than in the CA group; (2) patients with higher baseline levels of HBcAb, HBsAg, and ALB, and lower levels of fibrosis had depressed NK-cell-driven IFN-γ and TNF-α levels; and (3) a statistically predictive nomogram was created based on the Cox regression model tailored to individual patients to identify antiviral cytokines produced by NK cells in CHB.

The data presented in this study do not support the hypothesis that naïve patients have defective NK-cell IFN-γ production^35^. Analysis of cytokine production by circulating NK cells indicated that NK cells from CA patients with CHB infection displayed, on average, a superior ability to produce IFN-γ and TNF-α compared to those of CAN patients and healthy individuals. Nevertheless, 47.9% of CAN patients can be categorized into the group of high NK immunity of cytokine production by K-means analysis. This group of patients with high levels of antiviral cytokines seemed to be neither in an immune tolerant or an inactive state. Instead, they are more likely similar to the immune activate status and may benefit from antiviral therapy. This finding is consistent with the results from children and young adults during immunologic tolerance, which show higher antiviral immunity than healthy controls^3^. Therefore, ALT or HBV DNA levels are inadequate to reflect the presence/absence of an antiviral NK-cell response. Moreover, an active virus-specific immune response was detected in the liver in the absence of elevated ALT levels in both patients and animal models^5^.

We pooled the two patient groups to develop a model for the prediction of the NK-produced cytokine response to HBV. Although the model is based on data from patients enrolled with predefined inclusion and exclusion criteria, the generalizability of our results is probably good because of the large sample size and detailed, comprehensive analysis. With the strong association between several key parameters (Fibrosis, HBsAg, ALB, HBcAb) and the extent of NK-cell cytokine activity in the model, we then generated a nomogram that can be used to calculate the predicted likelihood of an NK-cell-produced cytokine response in an individual patient. With this model, we were able to categorize the patient populations in each clinical phase into two groups of high ( > = 0.361 NK score) or low NK cytokine function (<0.361 NK score). We observed that 45.31% of patients who were not recommended to receive treatment had high levels of antiviral cytokines produced by NK cells. CHB has been traditionally characterized into four phases, including the IT, IA (HBeAg positive), IA (HBeAg negative), and IC phases, reflecting the dynamic relationship between viral replication and evolution and the host immune response. However, some patients are not included in the above characteristic phases but are in a “grey zone (GZ)”. The data presented here showed that approximately 7.69%, 66.99%, 26.32%, and 44.83% of the patients in the IT, IA (HBeAg positive and negative), IC, and GZ phases, respectively, had high levels of NK-cell-specific cytokines. It should be noted that almost half of the CHB patients in the GZ group tended to have similar levels of cytokine production by NK cells as those for whom treatment is recommended. They possess the ability to produce NK-cell driven IFN-γ and TNF-α to counteract HBV infection. These observations illustrate the complexity of defining the host immune response to the virus.

In the current multivariable regression model, we found inverse associations between the levels of HBcAb, HBsAg, and ALB with cytokine production by NK-cells, while the fibrosis value had a significant positive association with cytokine expression. HBcAb is a serological marker, representing either the history or presence of HBV infection. The expression of HBcAb only is the most characteristic feature of occult HBV infection along with inactive immunity^36, 37^. HBcAb is considered to be accompanied by low levels of HBV DNA and a depressed immune reaction in the liver^38^. Similar to these results, our data implied that HBcAb is a strong indicator of NK cell-mediated immune quiescence. In addition to HBcAb, HBsAg and ALB were also identified as predictors of a lower antiviral response from NK-cell-produced cytokines. HBsAg is expressed by cccDNA-derived mRNA and released as a part of an infectious particle or non-infectious subvirion particles, the latter being produced independently of HBV replication^39^. Both *in vitro* and *vivo* studies showed that HBV proteins dysregulate cytokines, such as interleukin (IL)-10 or IL-18, which mediate cell signalling, to interfere with IFN-γ production by NK cells^40^. Thus, it is not surprising that patients with higher quantities of HBsAg express less antiviral cytokine by NK cells. However, higher fibrosis values were associated with increased cytokine expression by NK cells in the current patient population. It is well-known that hepatic stellate cells (HSCs) play a fundamental role in the development and pathogenesis of liver fibrosis^41–43^. The activation of HSCs and the transdifferentiation to myofibroblasts represent crucial paths towards fibrosis^44^. During the development of fibrosis, IFN-γ can be activated to exert its antifibrotic effect against the accumulation of HSCs in the liver^45^. Therefore, we proposed that the relationship between IFN-γ and fibrosis might be less significant in patients with advanced fibrosis or cirrhosis.

Since antiviral therapy for the treatment of CHB delays or prevents cirrhosis and reduces the incidence of HCC, its benefits are obvious. The questions of when to start treatment and flexibility of treatment initiation threshold have become more important and more controversial^5, 46^. It is widely assumed that waiting to initiate antiviral therapy until the occurrence of clinically active liver disease is an adequate standard of care. However, symptoms often are not apparent until a patient has terminal liver damage. One of the important reasons for not including other HBV-infected individuals beyond the current guidelines is the lack of immune activity or the presence of immunological tolerance in these patients. The viral burden is well-tolerated in these patients; thus, there is no indication for treatment. Rather, treatment is ill-advised because it may promote drug resistance^47^. The data presented in this study can identify HBV-infected individuals with high or low antiviral cytokine expression by NK cells, particularly HBV-specific T cells which are usually exhausted during native CHB infection. Higher IFN-γand TNF-α produced by NK cells has been demonstrated correlated to viral inhibition and HBsAg clearance^48–51^. In addition to direct antiviral effect, these cytokines could further regulate adaptive immune responses by activating T and B cells^22, 52, 53^. Some studies also uncovered that NK cells may play a detrimental role in HBV chronicity thorough killing HBV specific CD8^+^ cells. Such negative immunomodulatory functions were closely related to TNF-related apoptosis-inducing ligand (TRAIL) expressed on NK cells. Although TRAIL expression was correlated to the detrimental effect of NK cell against HBV-specific T cells, it could be reduced by antiviral therapy especially in those NUC treated patients after HBsAg clearance. The improved HBV-specific T cell functions were also observed at the meantime^33^. Collectively, patients could benefit from viral load reduction for increasing antiviral cytokine production and decreasing detrimental function of NK cells. Thus, we arbitrarily assumed that the proportion of patients in the CAN group with higher NK-cell cytokine function possibly can be the candidates of an earlier treatment intervention before irreversible liver damage occurs.

The strengths of this study include the following: (1) its novel information regarding the behaviour of NK cells in CHB patients regardless of whether they meet the treatment criteria. The patient population was reclassified into high or low activity of NK-cell cytokine production by K-means analysis. The demonstration that a certain number of subjects from the CAN group can produce high levels of NK-cell specific cytokines is direct evidence that these patients cannot be in a state of defective immunology. This observation illustrates the complexity of defining disease activity in CHB and the limitations of disease classification based on serology or biochemical markers alone. (2) The model could potentially be applied clinically to stratify CHB patients by their levels of efficient cytokines produced by NK cells. Those with high antiviral responses would be considered for protocols involving more frequent surveillance intervals and possibly antiviral strategies, while those with low responses would be triaged to less frequent surveillance protocols. However, our practical model is preliminary and we should include more patients to study in the future. And questions that remain unanswered by our study and merit future inquiry are as follows: (1) How does the cytokine activity in NK-cells change during CHB? A prospective longitudinal assessment would be desirable to identify the potential impact of disease fluctuations on cytokine composition and effector function. (2) Can our clinical prediction model of NK-cell specific cytokine be validated in a large cohort of NUC-treated patients? (3) Is the quantity of cytokines produced by intrahepatic NK cells correlated with or is regulated independently by peripheral NK-cell producing cytokines? (4) Ideally, large confirmatory groups would have been used for an external validation. An ongoing clinical trial may allow for further validation of the model in the near future.

## Conclusions

We have shown that almost half of the patients not meeting the treatment guidelines had high levels of NK-cell cytokine production, contrary to the current opinion of having either immune insufficiency or less liver injury. Moreover, IFN-γ and TNF-α can be incorporated into a clinical prediction model that can be used to stratify the relative antiviral response due to NK-cell cytokine production in CHB patients. Our immunological data provide a new argument to suggest that patients who preserve a high immune response to viral antigens may be suitable treatment candidates although they do not meet current treatment criteria.

## Electronic supplementary material

Supplementary material
