# Supplementary material for: A Practical Model Evaluating Antiviral Cytokines by Natural Killer Cells in Treatment Naïve Patients with Chronic Hepatitis B Virus Infection
Source: Sci Rep. 2017 Jul 19;7:5866. doi: 10.1038/s41598-017-06192-1 (PMC5517634; doi:10.1038/s41598-017-06192-1)

**A Practical Model Evaluating Antiviral Cytokines by Natural Killer Cells in Treatment Naïve Patients with Chronic Hepatitis B Virus Infection**

Xiaoyan Li^1^, Yurong Gu^1^, Xiaobo Guo^2,3,4^, Lin Gu^5^, Liang Zhou^1^, Xiaojuan Wu^1^, Xueqin Wang^2,3,6^，Zania Stamataki^7^, Yuehua Huang^1,5*^

^1^ Department of Infectious Diseases, The Third Affiliated Hospital of Sun Yat-sen University, Guangzhou, China.

^2^ Department of Statistical Science, School of Mathematics, Sun Yat-Sen University, Guangzhou, China.

^3^ Southern China Center for Statistical Science, Sun Yat-Sen University, Guangzhou, China.

^4^ Department of Ophthalmology, University of Melbourne, Melbourne, Australia

^5^ Guangdong Provincial Key Laboratory of Liver Disease Research, The Third Affiliated Hospital of Sun Yat-sen University, Guangzhou, China.

^6^ Zhongshan School of Medicine, Sun Yat-Sen University, Guangzhou, China

^7^ Institute for Immunology and Immunotherapy and NIHR Biomedical Research Centre, University of Birmingham, Birmingham, United Kingdom.

Xiaoyan Li, Xiaobo Guo and Yurong Gu contributed equally to this work.

*Corresponding author: Yuehua Huang

Correspondence:

Yuehua Huang, M.D., PhD

Department of Infectious Diseases & Guangdong Provincial Key Laboratory of Liver Disease Research, The Third Affiliated Hospital of Sun Yat-sen University. No.600 Tianhe Road. Guangzhou, China, 510630. Tel: 8620-85252702. Fax: 8620-85253305

Email: [huangyh53@mail.sysu.edu.cn](mailto:huangyh53@mail.sysu.edu.cn)

**Supplementary material**

**Supplementary Fig 1.** **Correlations of clinical virology parameters with percentages of NK^dim^ and NK^bright^ cells in CA patients.** Correlations of levels of HBV DNA, HBsAg, ALT and fibrosis, respectively, with frequencies of NK^dim^ (upper) and NK^bright^ cells (lower) in CA group patients. The *clinical* virology and r^2^ were shown.


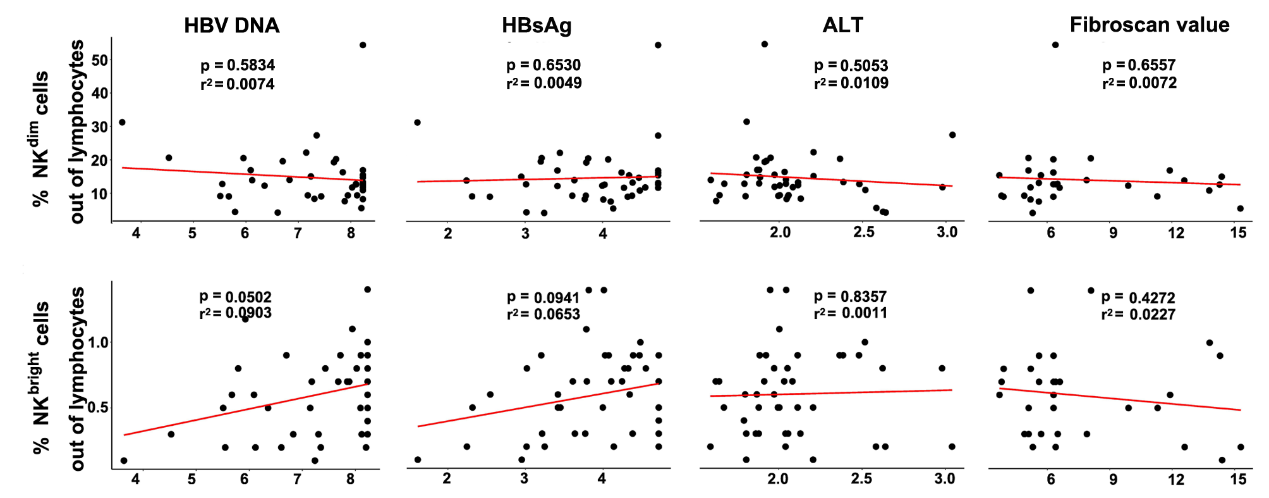


**Supplementary Fig 2.** **Correlations of clinical virology parameters with the percentages of NK^dim^ and NK^bright^ cells in CAN patients.** Correlations of levels of HBV DNA, HBsAg, ALT and fibrosis, respectively, with frequencies of NK^dim^ (upper) and NK^bright^ cells (lower) in CAN group patients. The *p* value and r^2^ were shown.


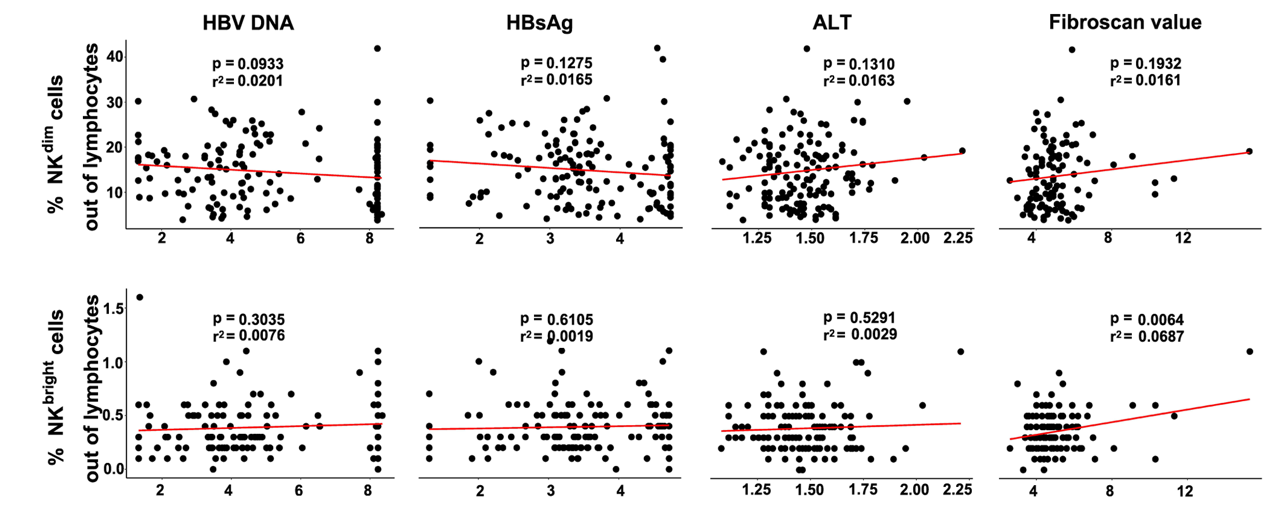


**Supplementary Fig 3**. **Correlations of clinical-virology parameters with NK-cell-driven cytokine expression in CA patients.** Correlations of clinical virology parameters with NK^dim^ cells expressing cytokines in CA patients. The *p*-value and r^2^ were shown.


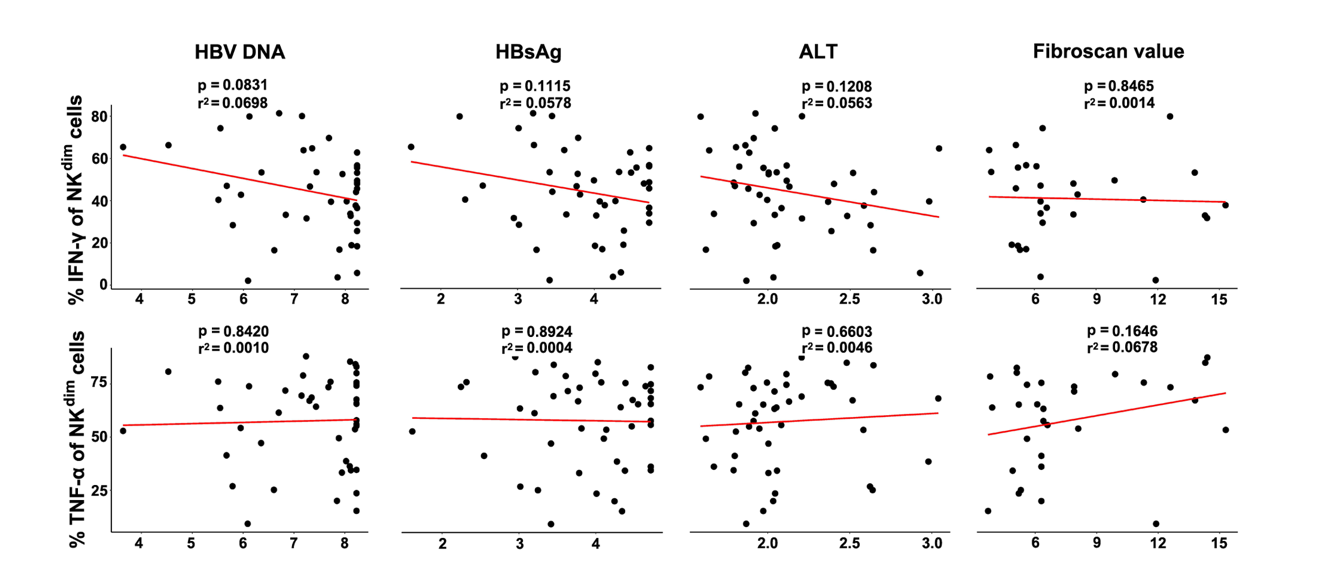


**Supplementary Fig 4.** **Correlations of clinical virology parameters with NK-cell-driven cytokine expression in CA patients.** Correlations of clinical virology parameters with NK^bright^ cells expressing cytokines in CA patients. The *p* value and r^2^ were shown.


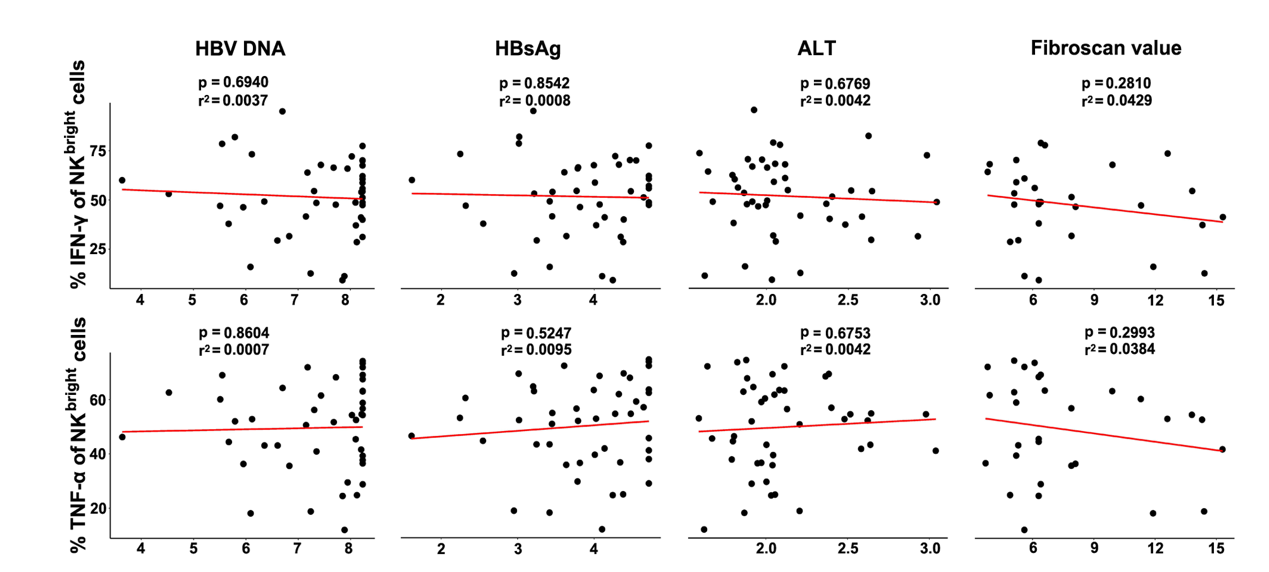


**Supplementary Fig 5**. **Correlations of clinical virology parameters with NK-cell-driven cytokine expression in CAN patients.** Correlations of clinical virology parameters with NK^dim^ cells expressing cytokines in CAN patients. The *p* value and r^2^ were shown.


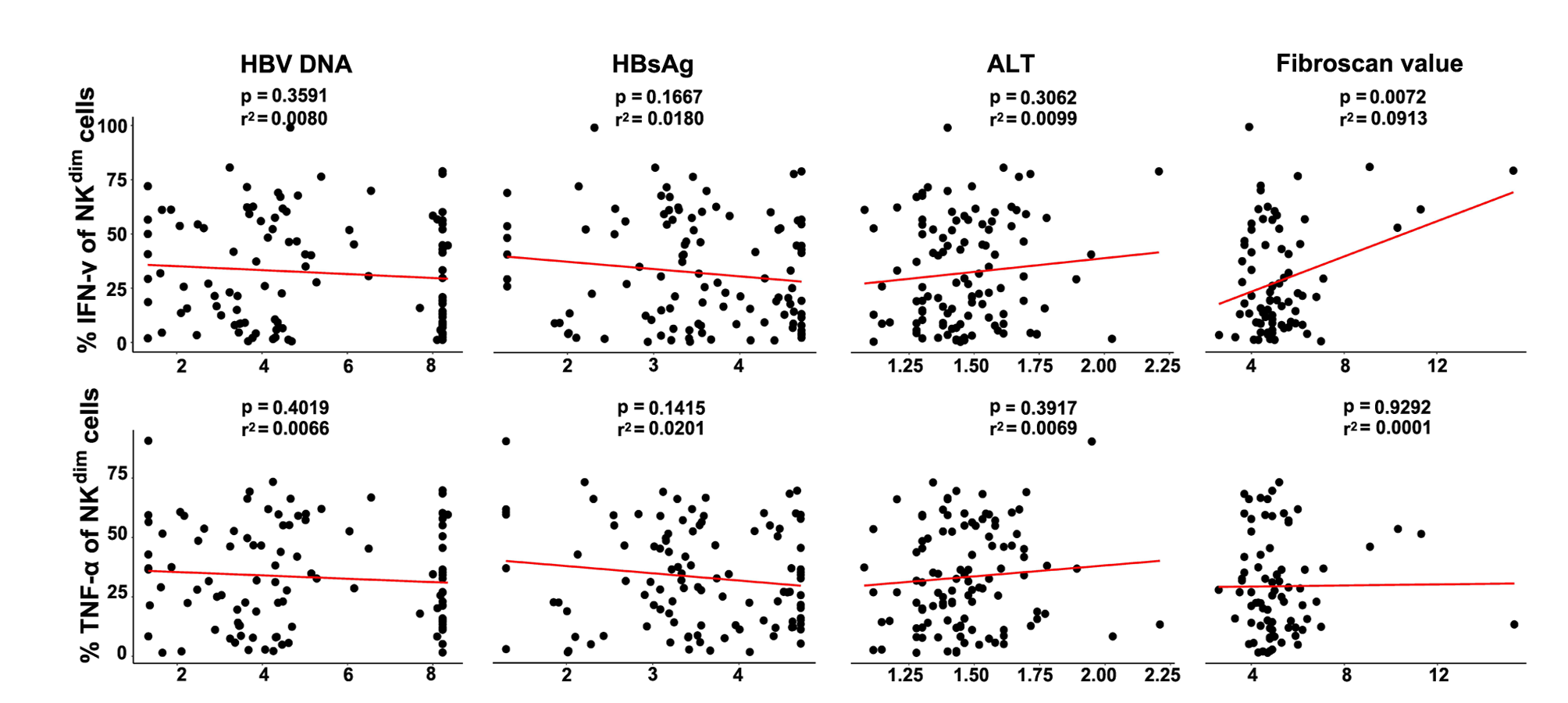


**Supplementary Fig 6.** **Correlations of clinical virology parameters with NK-cell-driven cytokine expression in CAN patients.** Correlations of clinical-virology parameters with NK^bright^ cells expressing cytokines in CA patients. The *p*-value and r^2^ were shown.


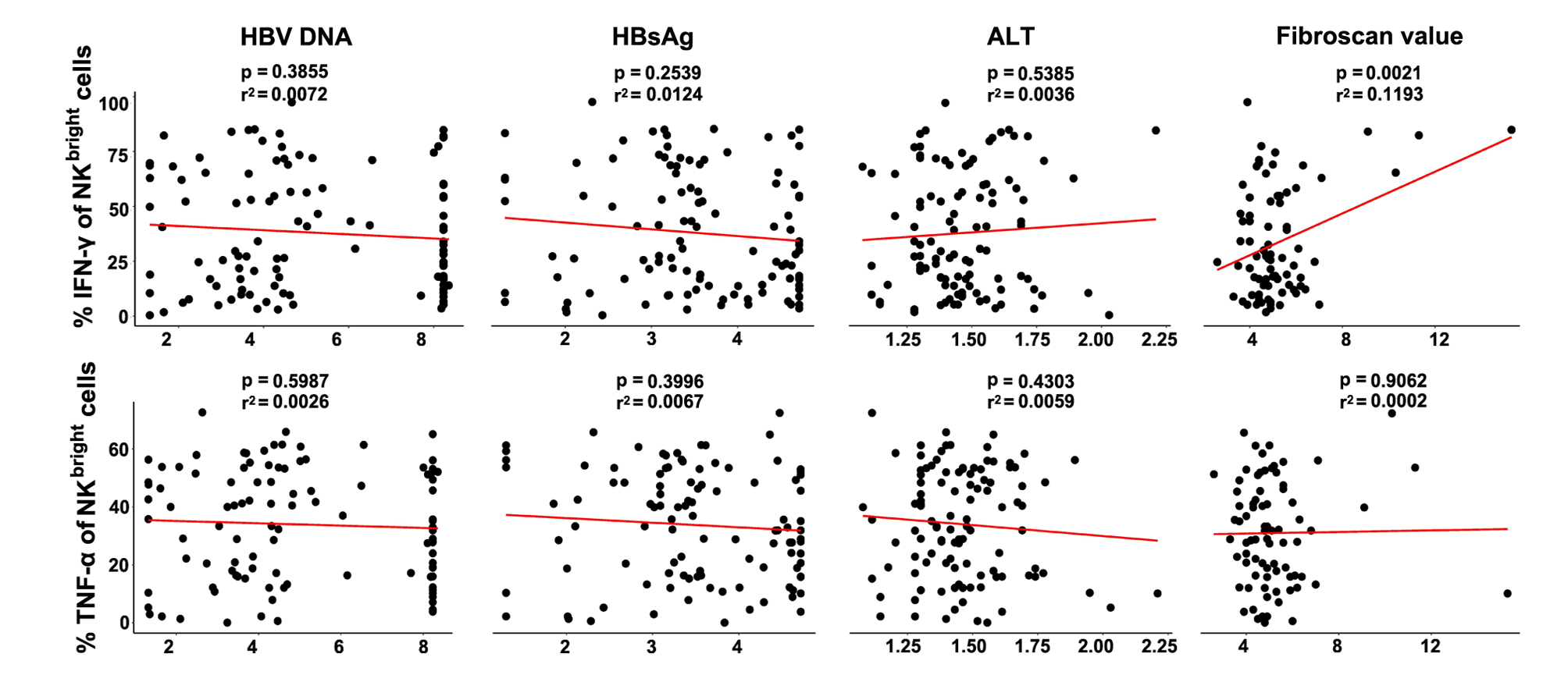

Supplement: Supplementary file 1 — Supplementary material [file 41598_2017_6192_MOESM1_ESM.docx]
